# Fine motor skills in adult Tourette patients are task-dependent

**DOI:** 10.1186/1471-2377-12-120

**Published:** 2012-10-11

**Authors:** Irene Neuner, Jorge Arrubla, Corinna Ehlen, Hildegard Janouschek, Carlos Nordt, Bruno Fimm, Frank Schneider, N Jon Shah, Wolfram Kawohl

**Affiliations:** 1 Department of Psychiatry, Psychotherapy and Psychosomatics, RWTH Aachen University, Aachen 52074, Germany; 2 Institute of Neuroscience and Medicine - 4, Forschungszentrum Juelich GmbH, Juelich 52425, Germany; 3 Department of General and Social Psychiatry, University of Zurich, Zurich, 8004, Switzerland; 4 JARA – Translational Brain Medicine, Aachen, Germany; 5 Department of Neurology, Section Neuropsychology, RWTH Aachen University, Aachen, 52074, Germany; 6 Department of Psychiatry Psychotherapy and Psychosomatics, RWTH Aachen University, Pauwelsstrasse 30, Aachen, 52074, Germany

## Abstract

**Background:**

Tourette syndrome is a neuropsychiatric disorder characterized by motor and phonic tics. Deficient motor inhibition underlying tics is one of the main hypotheses in its pathophysiology. Therefore the question arises whether this supposed deficient motor inhibition affects also voluntary movements. Despite severe motor tics, different personalities who suffer from Tourette perform successfully as neurosurgeon, pilot or professional basketball player.

**Methods:**

For the investigation of fine motor skills we conducted a motor performance test battery in an adult Tourette sample and an age matched group of healthy controls.

**Results:**

The Tourette patients showed a significant lower performance in the categories steadiness of both hands and aiming of the right hand in comparison to the healthy controls. A comparison of patients’ subgroup without comorbidities or medication and healthy controls revealed a significant difference in the category steadiness of the right hand.

**Conclusions:**

Our results show that steadiness and visuomotor integration of fine motor skills are altered in our adult sample but not precision and speed of movements. This alteration pattern might be the clinical vignette of complex adaptations in the excitability of the motor system on the basis of altered cortical and subcortical components. The structurally and functionally altered neuronal components could encompass orbitofrontal, ventrolateral prefrontal and parietal cortices, the anterior cingulate, amygdala, primary motor and sensorimotor areas including altered corticospinal projections, the corpus callosum and the basal ganglia.

## Background

Tourette syndrome (TS) is a neuropsychiatric disorder characterised by motor and phonic tics. Deficient motor inhibition underlying tics is one of the main hypotheses in its pathophysiology. Therefore the question arises whether this supposed deficient motor inhibition affects also voluntary movements. The severity of tics waxes and wanes over time. Patients often report that stress and teasing by others worsen tics, whereas focused activity such as reading or voluntary motor activities such as playing basketball reduces the occurrence of tics [1-4].

TS is often accompanied by comorbidities such as obsessive-compulsive disorder (OCD), depression and attention-deficit-hyperactivity disorder (ADHD) [5,6]. The cortico-striato-thalamo-cortical circuit plays an important role in the pathophysiology of TS. Tics respond to treatment with D_2_-receptor blocking agents with high striatal affinity [7-9]. Therefore, the neuroanatomy of tics has received particular attention in neuroimaging research on Tourette syndrome [10,11], highlighting a network of frontal areas, basal ganglia, insula and cerebellum, compatible with the notion that TS is the result of a failure in network maturation, particularly of the fronto-striatal-thalamic-cortical loop [12-14]. Structural changes have been described in Tourette patients, such as grey matter reduction in orbitofrontal, anterior cingulate and ventrolateral prefrontal cortices [15]. Alterations in amygdala structure and its functional connectivity are also reported [16-18].

Recent imaging studies in TS report an involvement of the corticospinal tract and of the underlying white matter under the supplementary motor area, the pre- and postcentral gyrus and the ventral-posterolateral nucleus of the right thalamus [19,20]. There are reports of reduced volumes of the caudate nucleus across the life span and thinning of sensorimotor cortices in proportion to tic severity in children suffering from Tourette syndrome [21]. Hypertrophy of limbic and prefrontal cortices and a smaller corpus callosum are associated with less pronounced tics in children with TS [22,23]. For the different clinical phenotypes of Tourette syndrome (e.g. simple tics, simple and complex tics, tics and OCD) Worbe and colleagues reported cortical thinning in primary motor regions in patients with simple tics. In patients suffering from simple and complex tics the cortical thinning was spread to larger premotor, prefrontal and parietal regions, and a trend for reduced cortical thickness in the anterior cingulated cortex and hippocampal morphology was reported in patients with comorbid obsessive-compulsive disorder [24].

Given the feature of tics as motor symptoms the question arises whether fine motor skills are affected in Tourette syndrome. There is a limited number of studies in children and adults to this end. In 1990 Bornstein published the results of a comprehensive neuropsychological test battery for 100 children and adolescents with Tourette syndrome [25]. T-values of the finger tapping task for the dominant and non-dominant hand were within normal limits. Performance for the grooved pegboard was normal for the dominant hand and impaired for the non-dominant hand (t=34.6). In a recent behavioural study in 11 adult Tourette patients Jonas and colleagues reported behavioural results on simple finger movements in adult Tourette patients with focus on echophenomena [4]. They described no significant difference in the reaction time of simple motor tasks between patients and a healthy controls group. However, when confronted with the task of copying a single finger movement following an incongruent biological stimulus Tourette patients showed a marked decline in performance with increased reaction times.

The clear affection of primary motor structures in recent neuroimaging studies would suggest that fine motor skills are impaired in Tourette patients [10,11,18,19,26-28]. On the other hand, clinical observations show that patients are able to suppress their tics at a certain degree of focused attention and also at some movements accomplishment, e.g. tics are often reported to be reduced in sports. Tourette patients are found among top athletes, professional piano players and neurosurgeons.

For the investigation of fine motor skills we conducted a motor performance test battery [Motorische Leistungsserie (MLS), Wiener Testsystem] in an adult Tourette sample. We aimed to address the following questions by our study:

a) Are fine motor skills in Tourette patients altered as possibly indicated by neuroimaging studies showing changes in the motor system?

b) What differences in task performance can be observed between TS patients and healthy controls?

## Methods

21 adult out-patients (4 female, 17 male, aged 18–48 years, mean age 30.9 ± 10.09 SD years) fulfilling the DSM-IV-TR criteria for Tourette syndrome and an age matched group of healthy controls (n=21, 4 female, 17 male, aged 18–48 years, mean 32 ± 9.7 SD years) participated in the study. In the Tourette patients group five patients suffered from obsessive-compulsive disorder (OCD), and two patients from attention-deficit-hyperactivity disorder (ADHD) according to DSM-IV guidelines. 13 out of 21 patients were currently under medication. Details of medication (current and lifetime) are listed in Table 1. A subgroup of the Tourette sample consisting of patients without comorbidities and without neuropsychiatric medication (n=10, 1 female, 9 male, aged mean 32.1 +− 11.6 years) was compared to the healthy controls to control for potentially confounding factors.

**Table 1:** Demographic and clinical data of Tourette patients (n=21)

**Subject**	**Age**	**Sex**	**OCD**	**ADHD**	**YGTSS global**	**YGTSS impairment**	**Current daily medication**	**Lifetime daily medication**
1	42	F	No	No	46	30	None	Tiapride up to 600mg
2	18	M	Yes	No	33	10	Sulpiride 100mg	Sulpiride 100mg
3	36	M	No	No	48	20	None	None
4	40	M	No	No	49	30	Tiapride 100mg	Tiapride 300mg
5	19	M	N0	No	21	10	None	None
6	19	M	No	No	43	20	None	Pimozide 2mg
7	24	M	No	No	37	10	None	None
8	37	M	Yes	No	57	30	Pimozide 4mg Citalopram 20mg	Pimozide 6mg Citalopram 20mg
9	21	M	No	Yes	56	30	Tiapride 400mg Methylphenidate 60mg	Tiapride 800mg Methylphenidate 60mg
10	38	M	No	No	37	10	None	None
11	25	F	No	Yes	43	20	Pimozide 2mg Methylphenidate 40mg	Pimozide 2mg Methylphenidate 40mg
12	23	M	No	No	24	10	None	None
13	22	F	No	No	37	20	Trimipramine 50mg	Trimipramine 50mg
14	24	M	No	No	49	20	None	None
15	27	M	No	No	47	20	Trimipramine 100mg	Trimipramine 100mg
16	27	M	Yes	No	55	30	None	None
17	28	F	No	No	47	20	Pimozide 2mg	Pimozide 2mg
18	47	M	Yes	No	69	30	None	None
19	48	M	No	No	37	20	None	None
20	48	M	No	No	53	30	None	Tiapride 100mg
21	35	M	Yes	No	48	20	None	Tiapride up to 400mg

(OCD = comorbid obsessive compulsive disorder, ADHD = comorbid attention deficit hyperactivity disorder, YGTSS = Yale Global Tic Severity Scale).

### Assessment

The study protocol included a standardized clinical interview according to AMDP (Association for Methodology and Documentation in Psychiatry) guidelines and detailed neurological evaluation by a board certified (neurology and psychiatry) physician (I.N.), physical examination, and an assessment with the Yale Global Tic Severity Scale (YGTSS) [29]. Psychiatric evaluation included assessment of comorbidities such as ADHD, OCD and depression according to DSM-IV-TR criteria. All patients were right handed according to the Edinburgh Handedness Test [30]. Control subjects were recruited from employees of the Research Centre Juelich and students from the RWTH Aachen University. In controls, intake of any medication, current psychiatric or neurological disorders or a history of any of them were exclusion criteria.

Clinical characteristics including Yale Global Tics Severity Scale (YGTSS), comorbidity and medication are listed in Table 1. The study was approved by the ethics committee of the RWTH Aachen University Clinic, and performed in accordance to the Declaration of Helsinki. All participants gave written informed consent.

**Figure 1:**
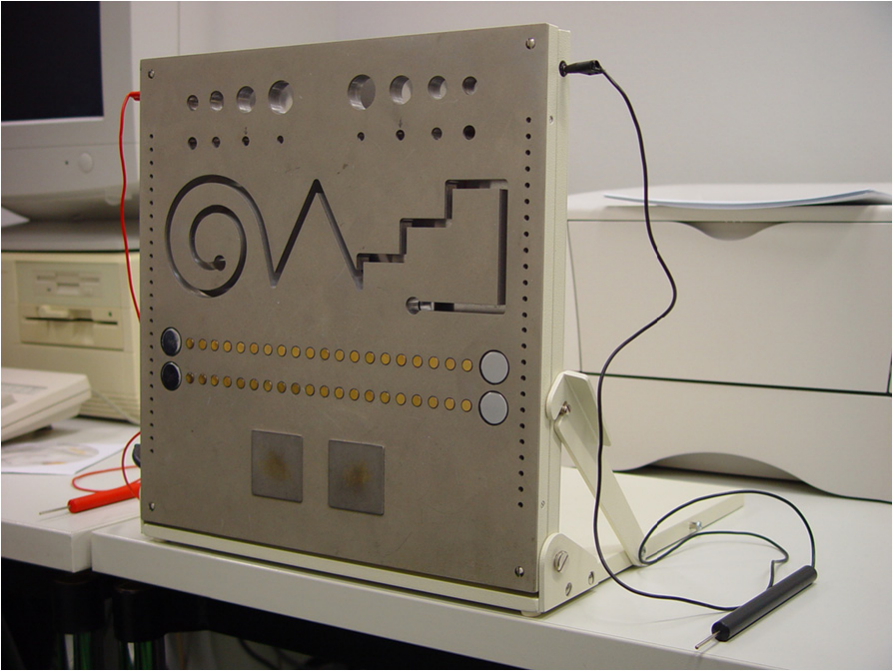
**Standardized pegboard of the MLS system.** For the subtests steadiness and contour copying the pegboard is presented in an upright position, for the subtests aiming and tapping in a horizontal position. For the subtest steadiness the participants inserted a stick in a hole (5.8 mm diameter, 32 seconds duration, top row, second one from centre) and were required to keep it there without touching by accident the lateral wall or back wall of the hole. The subtest contour copying requires the copying of a given trace without touching the lateral or back walls of the milled out trace (please see Figure 1 third row from top). The subtest aiming requires the volunteer to tap as fast and exact as possible along a line of 20 small circles (diameter 5 mm, distance between circles 4 mm) without touching the pegboard itself (fourth row of the pegboard in Figure 1). The subtest tapping consists of the instruction to tap on a rectangular plate (40mm one side) as fast and often as possible (quadratic area in the sixth row of the pegboard in Figure 1).

All subjects performed the S2-subform of the motor performance test battery (i.e. Motorische Leistungsserie, Wiener Testsystem) as described by Sturm and Buessing [31]. In short, it consists of 8 subtests, 4 for each hand. On a standardized pegboard the parameters steadiness, contour copying, aiming and tapping are assessed for both hands separately. For the subtests steadiness and contour copying the pegboard is presented in an upright position, for the subtests aiming and tapping in a horizontal position. Scoring for the subtests depends on accuracy as well as on speed with the exception of the steadiness subtest. Figure 1 shows a photograph of the standardized pegboard. For the subtest steadiness the participants inserted a stick in a hole (5.8 mm diameter, 32 seconds duration, top row, second one from centre) and were required to keep it there without touching by accident the lateral wall or back wall of the hole. The number of accidental contacts and duration of contacts (sec) were measured. This task tests for arm-head-steadiness.

The subtest contour copying requires the copying of a given trace without touching the lateral or back walls of the milled out trace (please see Figure 1 third row from top). It is assumed to test for the precision of arm and hand movements.

The subtest aiming requires the volunteer to tap as fast and exact as possible along a line of 20 small circles (diameter 5 mm, distance between circles 4 mm) without touching the pegboard itself (fourth row of the pegboard in Figure 1). It assesses the coordination between eye and hand within small distances.

The subtest tapping consists of the instruction to tap on a rectangular plate (40mm one side) as fast and often as possible (quadratic area in the sixth row of the pegboard in Figure 1). It measures the wrist-finger-speed.

The timing of each subtest and measurement of the correct and incorrect contacts with the pegboard is performed via the software program of the S2 – Test by Sturm and Büssing. It runs on a standard PC which is connected to the pegboard.

### Statistics

Kolmogorov-Smirnov-test was performed to control for normal distribution of the data. Data of the whole Tourette sample were compared to the data of the control group by means of a Mann–Whitney-U-test since no normal distribution had been revealed by the Kolmogorov-Smirnov-test. For statistical analysis a Bonferroni-Correction was performed. This conservative procedure has been chosen due to the large number of variables in a small sample size. The corrected p-level was p_Bonf_ ≤ 0.00625 because of the application of 8 subtests. YGTSS scores were correlated with the motor subtests using Pearson’s correlation coefficient. For evaluation of potential confounding influences by medication and comorbidities the subsample of Tourette patients without medication and without comorbidities was compared to the control group by Mann–Whitney-U-test. Aditionally, the medicated and non medicated subsamples of the Tourette-group were compared by Mann–Whitney-U-test.

## Results

### Results (t-values mean ± standard deviation) for the whole Tourette sample (n=21)

**Figure 2:**
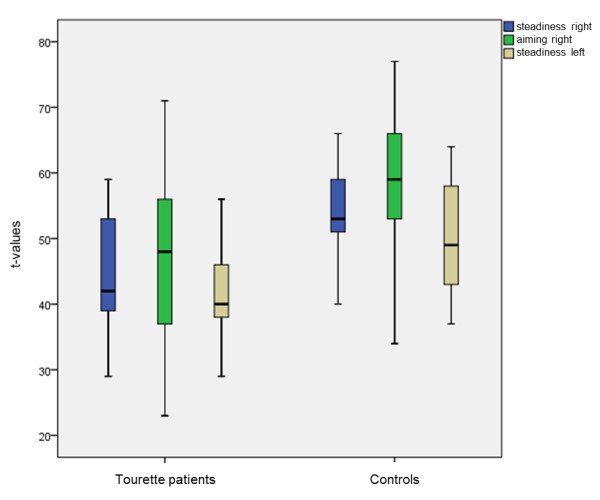
**Boxplots of the Bonferroni-significant t-values (steadiness right, aiming right, steadiness left).** Higher t-values indicate better performance.

The steadiness in the whole Tourette sample was 44.2 ± 8.5 for the right hand and 41.2 ± 7 for the left hand (See Figure 2). Copy of contours was 41.5 ± 7.5 for the right hand and 49.2 ± 11.3 for the left hand. Aiming was 45.7 ± 14.3 for the right hand (See Figure 2) and 50.4 ± 11.3 for the left hand. Tapping in the Tourette sample was 54.8 ± 8.6 for the right hand and 56.8 ± 11.4 for the left hand.

### Results (t-values mean ± standard deviation) for the control sample (n=21)

The steadiness in the control group was 53.9 ± 7.8 for the right hand and 50.8 ± 8 for the left hand (See Figure 2). Copy of contours was 45.9 ± 9.2 for the right hand and 53.9 ± 12.3 for the left hand. Aiming was 59.4 ± 10.4 for the right hand (See Figure 2) and 57.9 ± 12.9 for the left hand. Tapping was 59.8 ± 10.2 for the right hand and 59.9 ± 13.5 for the left hand.

### Results (t-values mean ± standard deviation) for the Tourette subsample without comorbidity and without medication (n=10)

The steadiness in the Tourette subsample without comorbidity and without medication (n=10) was 42.7 ± 7.8 for the right hand and 42.1 ± 7.1 for the left hand. Contour copying was 42.9 ± 6.2 for the right hand and 53.6 ± 12.2 for the left hand. Aiming was 50.3 ± 15.6 for the right hand and 52.2 ± 12.1 for the left hand. Tapping was 57.9 ± 8.8 for the right hand and 62.4 ± 9.1 for the left hand.

### Comparison of the whole Tourette sample (n=21) vs. controls (n=21)

After Bonferroni-correction, the performance in the subtests steadiness of the right hand (p=0.002), aiming of the right hand (p=0.002), and steadiness of the left (p=0.000) showed significant differences (p_Bonf_ ≤ 0.00625) between the patients and controls. Results are summarized in Table 2 for the whole Tourette sample versus age and sex matched controls.

**Table 2:** Fine motor skills in Tourette patients and controls, t-values

**Fine motor skills**	**t-values: mean ± SD TS-patients (n=21)**	**t-values: mean ± SD Controls (n=21)**	**p-values**
Steadiness R*	44.2 ± 8.5	53.9 ± 7.8	**.002**
Contacts by error			
**Copy contours R**	41.5 ± 7.5	45.9 ± 9.2	.057
Contacts by error			
Aiming R*	45.7 ± 14.3	59.4 ± 10.4	**.002**
Contacts outside target area			
**Tapping R**	54.8 ± 8.6	59.8 ± 10.2	.127
Hits in target area			
Steadiness L*	41.2 ± 7	50.8 ± 8	**.000**
Contacts by error			
**Copy contours L**	49.2 ± 11.3	53.9 ± 12.3	.158
Contacts by error			
**Aiming L**	50.4 ± 11.3	57 ± 12.9	.051
Contacts outside target area			
**Tapping L**	56.8 ± 11.4	59 ± 13.5	.351
Hits in target area			

Higher t-values indicate better performance. (R = right, L = left, *significant difference between Tourette patients and healthy volunteers (p_Bonf._< 0.00625), p_Bonf._ significant p-values in **bold**).

There was no significant correlation between the YGTSS total score or subscales.

### Comparison of Tourette subsample without comorbidity and without medication vs. healthy controls (n=10)

There was a significant difference for the item Steadiness R between the non- medicated patients without comorbidity and the healthy controls (p=0.002).

### Comparison of Tourette patients with medication (n=8) and without medication (n=13)

The comparison revealed no significant differences between the medicated and non-medicated subsamples.

## Discussion

In our sample of Tourette patients, following a conservative statistical method (Bonferroni-correction), the test battery consisting of steadiness, aiming, contour copying and tapping showed impaired motor skills in the subtest steadiness of the right hand, aiming of the right hand, and steadiness of the left hand. The analysis in the unmedicated subsample without comorbidities showed impairment in the subtest steadiness of the right hand.

### Subtest Steadiness

The subtest steadiness requires keeping the stick very stable in a small hole without touching the inner walls. It is supposed to test for arm-head-steadiness and proved to be a very challenging task for Tourette patients. Its key characteristic is the static position and the requirement not to modify the position once taken in any way. This finding is in good agreement with work reported by Heise and colleagues on altered modulation of intracortical excitability in movement preparation in TS [1]. They investigated motorcortical excitability at rest and during the preparation of a simple motor task via transcranial magnetic stimulation (TMS). The study showed motorcortical disinhibition in TS at rest. This is in good agreement with the hypothesis from Donald Cohen stating that Tourette is due to a deficit in inhibition [32]. In our fine motor skill assessment no open motor deficit was recognized during the performance of a task such as the contour copying. This clinical observation mirror the results from Heise and colleagues, which showed that TS patients who are to perform a voluntary motor task start from an abnormally disinhibited level of short-interval intracortical inhibition early during movement preparation with subsequent modulation of the inhibitory activity input similar to healthy volunteers [1]. Thus, one could hypothesize that ongoing movements requiring continuous moving and online adaptation, e.g. contour copying or tapping, reduce the level of motor inhibition and permit in this subtest a performance within the normal range. The task steadiness however lacks this continuous moving and online adaptation of movement and therefore proved to be a harder task for Tourette patients.

### Subtest Aiming

The subtest aiming analyses the coordination between eye and hand when working in small distances. This is impaired in Tourette patients with insufficient control over the dominant hand. The task requires precision and speed at the same time which puts Tourette patients under high pressure. Pressure in turn is known to increase the frequency of tics [2,3,16]. This finding is also in line with prior publications. They reported impairment on Tourette patients, particularly in tasks that require visuomotor integration [33,34]. Furthermore, it was described that motor ability in Tourette patients deteriorates with increasing difficulty [4,35,36]. This is in line with our findings of the subtest aiming.

### Subtest Tapping

In the subtest tapping, the tested wrist-finger speed was not impaired in Tourette patients. They performed as well as controls. There was no difference in speed. Also for this subtest the adaptation of motor inhibition towards a physiological level during movements as described by Heise and colleagues provides a good model for our finding [1].

### Pathophysiology

Given the four different types of motor tasks in our study the following two pathophysiological components within the motor system could be the source of the circumscribed deficits:

a) disinhibition at rest and in static tasks such as steadiness

b) deficits in visuomotor integration.

Starting from a status of disinhibition at rest, net inhibition is subsequently increased in order to achieve an adequate motor action [1]. Heise and colleagues discuss this as a compensatory mechanism where the motor cortex acts as a “relay station”, i.e. increasing inhibitory activation and thereby down-regulation neuronal excitability. So by performing e.g. the task contour copying or in daily life piano playing or drippling a basket ball the abnormal high neuronal excitability at rest in Tourette patients normalizes during the movement due to the increasing inhibitory signals from the motor cortex.

Neuroimaging results point on a pathophysiological level also to an influence of the frontal cortex. The analysis of the neuronal pattern of sequential finger tapping in an video-controlled fMRI task showed different neuronal activation patterns for the same resulting behavioural performance. One main finding was that with increasing task difficulty the incluence of frontal areas increased. This would be a good explanation of why Tourette patients perform within the normal limit in the fine motor skill tasks that demand constant motor adaptation and fine-tuning, in opposite to the task steadiness, in which no change in movement is required and therefore no change in the excitability of the system is demanded, as described by Heise and colleagues.

In our Tourette sample there was no correlation between the YGTSS subscores and the fine motor control. Thus, patients with a medium and a high tic score showed comparably fine motor skills. Therefore, an additional factor is assumed that is not mirrored by the YGTSS score and has a significant influence on motor performance. We hypothesize that this could be the degree to which patients are able to suppress their tics and to what degree focused attention decreases tic frequency. During the test battery, Tourette patients were focused on the tasks set and not distracted by other factors. On a pathophysiological level this would imply that via prefrontal input in the cortico-striato-thalamo-cortical circuit voluntary movements are modified. In an fMRI study investigating suppression of tics, Peterson and colleagues described recruitment of prefrontal areas and the anterior cingulate [26]. Kawohl and colleagues described activation in the anterior cingulate for tic suppression in a single-case study [37]. However, the different pathomechanisms compensate only partially since Tourette patients show clear deficits.

Another interesting point is the well-described abnormality of the structure and motor function of corpus callosum in Tourette patients [38]. The corpus callosum is a structure essential for bimanual coordination and unimanual lateralization, and both functions appeared to be impaired in Tourette patients [39]. These alterations found in Tourette patients, resulting from anomalous functional interhemispheric connectivity, might be reflected by the abnormality of the subtests steadiness bilaterally and aiming of the right hand.

### Clinical implications

Bloch and colleagues reported in a sample of children with TS a predictive role of the motor deficits with regard to glocal social functioning and tic severity (34). They investigated a cohort of 32 children twice, once 8–14 years old and a second time in average 7.5 years later. In this cohort poor performance with the dominant hand on the Purdue Pegboard test was associated with worse adulthood tic severity. Negative results of the Beery Visual-Motor Integration test and the Purdue Pegboard test also predicted worse adulthood gobal functioning (Bloch et al. 2006 (is listed already as reference 34). Our sample differs in two important aspects from Bloch’s cohort. One point is that we investigated adult Tourette patients and the other that we performed the study at one time point. However, our adult Tourette patients – so Tourette patients in whom tics persisted over childhood - present fine motor skill deficits. In this regard our data are complementary to Bloch’s. From a clinical perspective the finding that fine motor skills are task-dependent might help in counselling patients and their families with regard to professional choices.

### Limitations and outlook

Out of our findings arise further questions for future studies and point to limitations in our current study. Noteworthy is that in the subsample without medication and comorbidities only steadiness right –dominant hand in all patients– was significantly impaired in comparison to healthy controls. In the whole group steadiness right and left and aiming right was impaired. This on one hand underlines the robustness of the finding steadiness right, and on the other hand raises the question about lateralization of movement impairment in Tourette patients. On the basis of the well described alterations in the corpus callosum in children and adults [19,21,23], the sample size needs to be further increased and bimanual tasks needs to be added to the MLS test battery to disentangle possible lateralization effects from insufficient statistical power. Also, a longitudinal design with assessment before and on medication would shed light on the role of medication on the excitability of the motor system in Tourette and hence the fine motor skills [40]. Whether and to what degree comorbidities influence fine motor skills in Tourette would also require a larger sample, however the impaired steadiness on the right side seems to be a robust finding. One could also consider video-tapeing of the patients during the motor assessment as e.g. Jonas and colleagues did in their sample [4]. The positive aspect would be to analyse possible effects of tics and tic suppression on the movement performance, the negative aspect could be to increase the pressure on the patients which in turn might modify the results due to a possible increased tic frequency under additional pressure through the video-taping.

## Conclusions

In summary, our results show that steadiness and visuomotor integration of fine motor skills are altered in our adult sample but not precision and speed of movements. This alteration pattern might be the clinical vignette of complex changes and adaptations in the excitability of the motor system in Tourette syndrome. The motor output underlies complex interactions from cortical and subcortical structures which are known to be structurally and functionally altered in Tourette. Based on neuroimaging studies, the potentially underlying neuronal network encompasses the orbitofrontal and ventrolateral prefrontal cortices, parietal cortices, the anterior cingulate, the amygdala, primary motor and sensorimotor areas and their altered corticospinal projections, the corpus callosum and the basal ganglia [15,17,19,20,24,38,39,41-43].

## Competing interests

Dr. Janouschek, Mrs. Ehlen, Dr. Nordt, Dr. Neuner, Dr. Fimm, Mr. Arrubla have no conflict of interest to declare. Prof. Schneider received compensation as a consultant for Janssen-Cilag, AstraZeneca, and Otsouka. Prof. Schneider received compensation for scientific talks or contributions in a prize jury by Janssen-Cilag, Wyeth, and AstraZeneca. Prof. Schneider received funding for investigator initiated projects from AstraZeneca, Lilly and Pfizer. Prof. Shah acknowledges funding from the BMBF Germany and Siemens, Germany for the 9.4T project. Prof. Kawohl received compensation as a consultant for Janssen-Cilag. Prof. Kawohl received compensation for scientific talks by Eli Lilly, Bristol-Myers Squib and Vifor.

## Authors’ contributions

1) Research project: Conception: IN, FS, Organization and Execution: IN, CE, BF, NJS 2) Statistical Analysis: CN, WK, HJ 3) Manuscript IN, JA, HJ, FS, WK, N.S. All authors read and approved the final manuscript.

## Pre-publication history

The pre-publication history for this paper can be accessed here:

http://www.biomedcentral.com/1471-2377/12/120/prepub
